# Reward value shapes the time course of self-bias

**DOI:** 10.3758/s13421-025-01763-4

**Published:** 2025-08-04

**Authors:** Parnian Jalalian, Marius Golubickis, Yadvi Sharma, Rinki Kanakraj, Esther S. Selvaraj, C. Neil Macrae

**Affiliations:** 1 School of Psychology, University of Aberdeen, King’s College, King’s College, Aberdeen, AB24 3FX Scotland UK; 2 Department of Cognitive Sciences, United Arab Emirates University, Al Ain, United Arab Emirates

**Keywords:** Self, Ownership, Self-prioritization, Reward value, Time course

## Abstract

Personal possession wields a potent influence on decision-making, such that objects owned-by-self are categorized more speedily than comparable items belonging to other people. The temporal characteristics of this self-prioritization effect, however, are little understood. Notably, it is uncertain whether the duration of self-bias is sensitive to the characteristics of owned objects, particularly when the items have implications for the self-concept. Accordingly, using an object-classification task, here we explored the extent to which a theoretically important stimulus-related factor—reward value—influences the time course of self-bias. Across three experiments, participants classified high- and low-value items (i.e., stones [Exp. 1], cryptocurrencies [Exp. 2 & 3]) that allegedly belonged to self and a friend. A consistent pattern of effects was observed, indicating that reward value moderated both the extent and persistence of self-prioritization. Specifically, although self-prioritization emerged for both high- and low-value items, this facilitatory effect was larger and more enduring for the former (vs. latter) stimuli. These findings highlight the dynamic character of decisional bias, with implications for theoretical accounts of self-function.

**Supplementary Information:**

The online version contains supplementary material available at 10.3758/s13421-025-01763-4.

A fascinating aspect of the self-concept is that its contents extend beyond body and mind to include, amongst other things, one’s personal belongings. These items are noteworthy for good reason. As Belk (1991) observed, “Possessions can give us a sense of who we are, where we have come from, and where we are going” (p. 37). Unsurprisingly, therefore, ownership exerts a powerful influence on cognition and action (Morewedge, 2021). As extensions of the self, one’s chattels are believed to be more prized and desirable than identical items that belong to other people (Beggan, 1992; Belk, 1988, 1991; Kahneman et al., 1991; Knetsch & Sinden, 1984; Morewedge & Giblin, 2015). Additionally, personal possession enhances the detectability, learning, and memorability of objects, even when the items in question are inconsequential and have been randomly assigned to self (Constable et al., 2011, 2014; Cunningham et al., 2008, 2013; Englert & Wentura, 2016; Golubickis et al., 2018, 2019, 2023; Golubickis & Macrae, 2022; Jalalian et al., 2023, 2024a); Lockwood et al., 2018; Sparks et al., 2016). Affirmed by an extensive literature, ownership facilitates information processing and response selection.

In capturing the effects of personal possession on decision-making, recent empirical endeavours mirror a parallel line of inquiry that has considered how self-relevance influences the ease with which stimuli linked with various social targets (e.g., self, friend, stranger) can be identified (Humphreys & Sui, 2016; Sui & Humphreys, 2015, 2017). The findings have been unequivocal. Absent the familiarity confounds that limited earlier investigations using faces and forenames as the stimuli of interest (Bargh & Pratto, 1986; Gray et al., 2004; Keyes & Brady, 2010; Shapiro et al., 1997), self-associated items—including geometric shapes, abstract symbols, colours, sounds, and avatars—are given precedence during decisional processing, the aptly named self-prioritization effect (Caughey et al., 2021; Falbén et al., 2020; Golubickis et al., 2017, 2020, 2021b; Golubickis & Macrae, 2021; Keil et al., 2023; Macrae et al., 2017, 2018; Schäfer et al., 2015, 2016; Sui et al., 2012, 2015; Svensson et al., 2022, 2023; Wang et al., 2016; Woźniak & Knoblich, (Woźniak, and Knoblich, 2019); Yin et al., 2019). Like one’s personal possessions, even arbitrary stimuli profit from self-association (Humphreys & Sui, 2016; Sui & Humphreys, 2015, 2017).

## Ownership and decisional bias

Notwithstanding the potent facilitatory influence that ownership wields on decisional processing, stimulus prioritization is by no means an inevitable outcome. Rather than reflecting a mandatory facet of decision-making, self-prioritization is better characterized as a conditionally automatic phenomenon, emerging only under certain conditions (Golubickis & Macrae, 2023). In this respect, several task- and stimulus-related factors have been shown to influence the effects of personal possession on the generation of self-bias (Constable et al., 2019; Dalmaso et al., 2019; Falbén et al., 2019; Jalalian et al., 2023; Siebold et al., 2015; Stein et al., 2016; Svensson et al., 2022; Woźniak & Knoblich, (Woźniak et al., 2022)). For example, unless establishing the personal relevance of stimuli is the instructed task at hand, self-prioritization fails to occur (Constable et al., 2019; Dalmaso et al., 2019; Falbén et al., 2019; Macrae et al., 2017; Stein et al., 2016; Woźniak & Knoblich, (Woźniak et al., 2022)). Similarly, when items can boost or threaten the self-concept—as is the case with objects that vary in valence—only positive (vs. negative) stimuli trigger self-prioritization (Golubickis et al., 2021a, b; Hu et al., 2020; Lee et al., 2023; Vicovaro et al., 2022). Thus, although exerting substantial influence, the effects of self-association are by no means compulsory.

Despite growing understanding of the conditions under which ownership impacts decision-making, unanswered issues nevertheless remain. Conspicuous among these are questions pertaining to a central yet largely unexplored aspect of self-bias—its temporal characteristics (Jalalian et al., 2024a). If, as has widely been reported, self-prioritization is a core product of decision-making, then one would anticipate the advantages of personal possession to be lasting (Humphreys & Sui, 2016; Sui & Humphreys, 2015, 2017). That is, self-prioritization should be a persistent behavioural effect. Whether this is in fact the case, however, has yet to be established (Haciahmet et al., 2023; Lu et al., 2024; Sui et al., 2023; Svensson et al., 2022). Muddying the waters is the method that has been used to index the emergence of self-bias. As an illustrative case in point, take the self-prioritization effect reported by Golubickis et al. (2018). In a task context in which self and friend ostensibly possessed either pens or pencils, participants were required to classify 224 stimuli (112 pens & 112 pencils) in terms of their assigned owner (i.e., owned-by-self vs. owned-by-friend). Mean response times were then computed and compared for self-owned and friend-owned items, with the results revealing faster responses toward the former (vs. latter) objects (i.e., self-prioritization; Falbén et al., 2019, 2020; Golubickis et al., 2019; Golubickis & Macrae, 2021).^1^

Although revealing decisional bias, the strategy of aggregating performance on target-related items (i.e., self vs. friend) across the entire testing session yields little insight into the time course of self-prioritization. For example, are the benefits of self-association a stable property of decisional processing or do they fluctuate as the object-classification task progresses? Relatedly, to what extent do characteristics of the to-be-judged stimuli influence the temporal profile of self-bias? Addressing these issues is important for several reasons. As noted previously, a dominant viewpoint is that self-prioritization is a mandatory product of decisional processing (Humphreys & Sui, 2016; Sui & Humphreys, 2015, 2017). If, therefore, self-bias was shown to vary over time, this position would merit further scrutiny and modification (Constable et al., 2019; Dalmaso et al., 2019; Falbén et al., 2019; Woźniak & Knoblich, (Woźniak et al., 2022)). Additionally, by identifying stimulus-related characteristics that temper the persistence of self-prioritization, research has the capacity to enhance understanding of exactly where and when personal possession is likely to exert an enduring (vs. transient) influence on decision-making.

To date, research investigating the temporal characteristics of self-prioritization has been noticeably absent, with only a single study considering this matter in detail. Employing a standard shape-label matching task in tandem with a temporally sensitive analytical strategy (i.e., a moving average approach), Jalalian et al. (2024a) showed that self-prioritization was a stable product of decisional processing. That is, response latencies were faster to self-associated compared with either friend-associated or stranger-associated stimuli across the entire testing session. Crucially, however, this stability may have derived from a procedural aspect of the experimental paradigm that was adopted. It is possible, for example, that Jalalian, et al., (2024a) demonstration of enduring self-bias was a consequence of associations being forged between social targets (i.e., self, friend, stranger) and stimuli that had no inherent significance other than their salience in the immediate task context (Humphreys & Sui, 2016; Sui & Humphreys, 2015, 2017). It therefore remains to be seen whether similar effects would arise when the items in question are nontrivial and have implications for the self-concept (Golubickis et al., 2021a; Hu et al., 2020; Lee et al., 2023; Vicovaro et al., 2022). Under such conditions, it is possible that persistence may not be a characteristic of self-bias.

## Reward value and self-prioritization

A fundamental stimulus property with the potential to shape the time course of self-prioritization is reward value (i.e., stimulus desirability). Indeed, how exactly self- and reward-based processing are related has been a productive line of inquiry (Lee et al., 2023; Northoff & Hayes, 2011; Sui et al., 2012, 2016, 2023; Sui & Humphreys, 2015; Wang et al., 2021; Yankouskaya et al., 2020, 2023). Closely interwoven, reward value and salience are critical variables in attention, associative learning, and decision-making. Reward value stems from different predicted outcomes; rewards and punishments. Whereas stimuli associated with rewards are positively valued and approached, items associated with punishments are negatively valued and avoided. The salience of stimuli derives from the magnitude of predicted outcomes (i.e., value-driven salience), such that items associated with increasingly positive or negative consequences attract greater attention, thereby facilitating task performance (e.g., faster response times; Anderson et al., 2011; Failing & Theeuwes, 2018; Kim et al., 2021).

Work on value-based processing has direct implications for the temporal dynamics of self-bias. Consider, for example, a task setting in which self and friend own objects that vary in relative positivity (e.g., self and friend own both high- and low-value items). Given differences in reward value—hence, motivational salience and implications for the self-concept—one would expect self-prioritization to emerge for both high- and low-value objects, but for this facilitatory effect to be larger and more enduring for the former stimuli (Sui et al., 2016, 2023; Yankouskaya et al., 2020, 2023). That is, the self-enhancing qualities of high-value (vs. low-value) possessions should increase both the extent and duration of decisional bias (Alicke & Sedikides, 2009; Golubickis at al., 2021a; Ye & Gawronski, 2016).

Although not formally addressing the time course of self-bias, an identical prediction can be derived from the influential Self-Attention Network (SAN) model (Humphreys & Sui, 2016; Sui & Humphrey, (Sui et al., 2015)). According to this account, via bidirectional connections between regions of the prefrontal (i.e., ventromedial prefrontal cortex [vMPFC]) and temporal (i.e., posterior superior temporal sulcus [pSTS]) cortices, attentional operations increase the social salience of self-relevant material, with attendant benefits during stimulus appraisal and decision-making (Sui et al., 2012, 2013). Of relevance to the current investigation, neural activity related to value-based decision-making has also been reported in the vMPFC (Behrens et al., 2007; Clithero & Rangel, 2014; Rushworth et al., 2011). Reflecting sensitivity to the value of predicted outcomes, this prefrontal activity contributes to the increased salience of stimuli associated with larger rewards (Bartra et al., 2013; Strait et al., 2014). In the context of self–other decision-making, this suggests that high-value stimuli should produce larger and more enduring self-prioritization effects than their low-value counterparts.

## The current research

Using an object-classification task (Golubickis et al., 2018, 2019, 2021b), here we considered how reward value influences the temporal profile of self-prioritization. In three experiments, participants classified high- and low-value items—with either established (e.g., stones, Exp. 1) or assigned (e.g., cryptocurrencies, Exp. 2 & 3) worth—that allegedly belonged to self and a friend. To examine the temporal characteristics of self-prioritization, following Jalalian, et al. (2024a), a moving average analytical approach was adopted (Johnston et al., 1999). It was expected that self-prioritization would be impacted by reward value, such that the benefits of personal possession would be more pronounced and persistent for high-value compared with low-value objects (Sui et al., 2016, 2023; Yankouskaya et al., 2020, 2023).

## Experiment 1: Owning stones

### Method

#### Participants and design

Forty participants (19 women, 21 men: *M*_age_ = 23.77 years, *SD* = 2.95), with normal or corrected-to-normal visual acuity, completed the experiment. Data were collected online using Prolific Academic (http://www.prolific.co), with each participant receiving compensation at the rate of £10 (~$13) per hour. Informed consent was obtained from participants prior to the commencement of the experiment and the protocol was reviewed and approved by the Ethics Committee at the School of Psychology, University of Aberdeen. The experiment had a 2 (owner: self vs. friend) × 2 (object value: high vs. low) repeated-measures design. Based on previous research (Golubickis et al., 2018, 2021a), an a priori power calculation for an Owner × Object Value interaction indicated that 40 participants with a single repetition afforded 87% power to detect a medium-to-large effect size (*d* = 0.65, var(error) + var(P*O*V) = .417; PANGEA, Version 0.0.2).

#### Stimulus materials and procedure

The experiment was conducted online using Inquisit software, which participants accessed via a web link. Participants were informed the experiment comprised a categorization task featuring four objects: sapphires, rubies, marbles, and pebbles (Golubickis et al., 2018, 2019). Images of the stimuli were then shown on the computer screen, and participants were told that, before the task commenced, the computer would randomly assign two objects to be owned by self (i.e., self-owned) and two objects to be owned by their best friend (i.e., friend-owned). At this point, each participant provided the forename of their best friend and object assignment took place (i.e., text on the computer screen revealed which objects were self-owned and which were friend-owned). Crucially, self and friend were each assigned a high- (i.e., sapphires or rubies) and a low-value (i.e., marbles or pebbles) item, with object assignment counterbalanced across the sample.

**Fig. 1:**
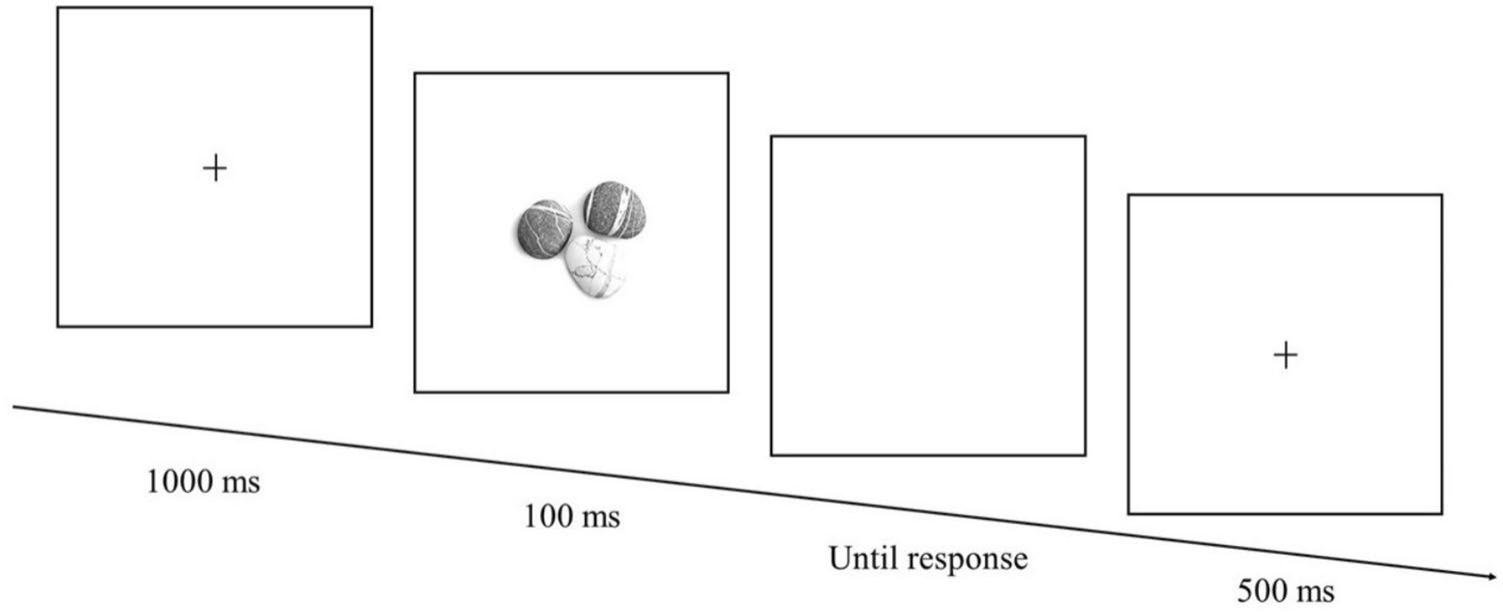
Example of an experimental trial

Next, participants were told they would be presented with individual pictures of the objects. Their task was to indicate, as quickly and accurately as possible, who owned the items (i.e., owned-by-self vs. owned-by-friend). Four pictures were used, with each containing three exemplars from the relevant category (e.g., three pebbles; see Fig. 1). Responses were given using two keys (i.e., N & M), with the meaning of the keys counterbalanced across participants. Each trial commenced with the presentation of a central fixation cross for 500 ms, followed by an image of the objects (220 × 220 pixels in size) which remained on the screen for 100 ms. If a response was not provided within 3,000 ms, the next trial commenced. The intertrial interval was 500 ms. Following 12 practice trials, three blocks of 100 trials (i.e., 300 trials in total) were completed. On finishing the task, participants were debriefed and thanked.

### Results

#### Aggregated analysis

Four (two women, two men) participants failed to follow the experimental instructions (i.e., responded with random button presses), thus were excluded from the analysis. Responses faster than 200 ms and slower than 1,100 ms were also omitted, which eliminated less than 2% of the total number of trials (Golubickis et al., 2018). A Shapiro–Wilk test indicated that reaction times (RTs) were normally distributed (*W* = .974, *p* = .531). As such, together with response accuracies, participants’ mean correct RTs were submitted to a 2 (owner: self vs. friend) × 2 (object value: high vs. low) repeated-measures analysis of variance (ANOVA). Analysis of the RTs revealed a main effect of owner, *F*(1, 35) = 14.85, *p* < .001, η_p_^2^ = .30, and a significant Owner × Object Value interaction, *F*(1, 35) = 5.95, *p* = .02, η_p_^2^ = .14 (see Table 1). Follow-up analyses yielded a significant self-prioritization effect for high-value objects, *t*(35) = 4.07, *p* < .001, *d*_z_ = 0.68, such that responses were faster to self-owned compared with friend-owned items. No such significant effect was observed for low-value objects, *t*(35) = 1.02, *p* = .314, *d*_*z*_ = 0.17.

**Table 1:** Mean RT (ms) and accuracy (%) as a function of owner and object value (Exp. 1)

	Object value	
Owner	High	Low
*RT*		
Self	488 (67)	503 (64)
Friend	517 (76)	508 (66)
*Accuracy*		
Self	96 (4)	93 (8)
Friend	93 (7)	95 (6)

Standard deviation in parentheses.

Analysis of response accuracies revealed only a significant Owner × Object Value interaction, *F*(1, 35) = 8.57, *p* = .006, η_p_^2^ = .20 (see Table 1). Follow-up analyses indicated that, for high-value objects, responses were more accurate to self-owned compared with friend-owned items, *t*(35) = −2.72, *p* = .010, *d*_z_ = 0.45. For low-value objects, this effect was reversed, *t*(35) = 2.30, *p* = .027, *d*_z_ = 0.38.

#### Moving window analysis

**Fig. 2:**
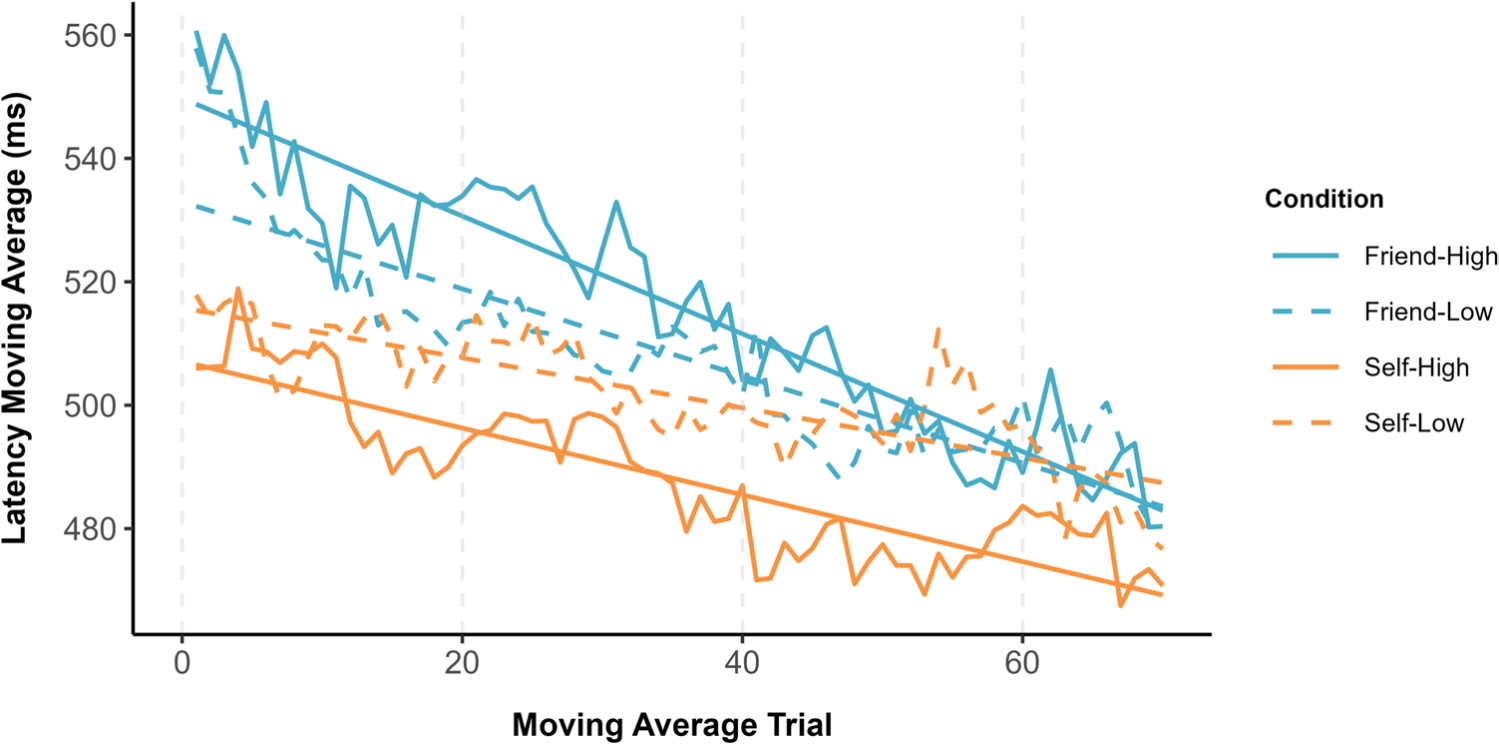
Moving window latency averages as a function of owner and object value

To explore the time course of self-bias, following Jalalian et al. (2024a), a moving average approach (i.e., window size = 10 trials) was applied to the RTs. A window size of 10 captured approximately 13% of the total number of trials and is consistent with the practice of selecting a moving window size between 5% and 20% of the time series (Chatfield, 2003). Initially, trials were renumbered to create a unique identifier for each participant as a function of the experimental conditions. Next, with a specified window size of 10 trials, a moving average of response latencies across correct trials was calculated. Moving averages were computed using the ‘rollapply’ function from the *zoo* package (Zeileis & Grothendieck, 2005), configured to compute the mean latency within each window while omitting missing values. The alignment parameter was set to ‘center’, meaning that the moving average at each point was established using data from both preceding and succeeding trials within the window. This analytical approach was adopted to smooth short-term variability in response latencies (see Fig. 2). To establish that the current results were not a consequence of the selected window size, additional analyses were undertaken using window sizes of five and 15 trials (capturing approximately 7% and 20% of the data, respectively; see Supplementary Information). The results observed in these additional analyses were consistent with those obtained when a window size of 10 trials was applied.

Linear models were fitted to explore the relationship between trial number and moving window latency averages, segmented as a function of experimental condition. This modeling approach describes how the RTs developed across trials, identifying trends within each experimental condition. The intercept in a linear model represents the expected value of the dependent variable (i.e., latency moving average) when all the independent variables (i.e., renumbered trial numbers) are set to zero. As such, it specifies the starting point of the moving average in each experimental condition before the task has commenced, thus providing a baseline against which the effect of trial progression on response latencies can be compared. In so doing, it informs understanding of the initial state of cognitive processing or response readiness at the beginning of task. The analysis also estimated the slope of the models to reveal the speed at which response latencies changed as the task unfolded. The determination coefficient (*R*^2^) was adopted to determine the proportion of variance in latency moving averages that was accounted for by the model, reflecting the strength of the relationship between trial progression and latency (overall, 78.3% of the variance was explained).

The intercepts and slopes derived from the linear models were submitted to a 2 (owner: self vs. friend) × 2 (object value: high vs. low) repeated-measures ANOVA. Analysis of the intercepts revealed only a main effect of owner, *F*(1, 35) = 22.25, *p* < .001, η_p_^2^ = .39, such that the starting point of the moving average was faster for self-owned (*M* = 512 ms, *SE* = 12 ms) compared with friend-owned (*M* = 544 ms, *SE* = 12 ms) items. Similarly, the analysis of slope coefficients again yielded only a main effect of owner (*F*(1, 35) = 11.58, *p* = .002, η_p_^2^ = .25, indicating that the rate at which the moving average decreased over trials was higher for friend-owned (*M* = −.88, *SE* = .16) compared with self-owned (*M* = −.47, *SE* = .16) objects.

To further investigate the temporal dynamics of self-prioritization, additional cluster analyses were undertaken. These focused on the comparison between the moving averages for self-owned and friend-owned items for each object value condition (Jalalian et al., 2024a). First, for each trial, a paired-sample *t* test was conducted to establish the presence of a significant self-prioritization effect (i.e., RT_self_ < RT_friend_). This yielded a dataset of *p* values, with each representing the probability of observing a latency difference by chance. These *p*-values were then corrected for a false discovery rate using the Benjamini-Hochberg procedure (*fdr* correction method on p.adjust; Benjamini & Hochberg, 1995). This correction is necessary as it minimizes the risk of Type I errors. Clusters were identified based on contiguous trials displaying significant latency differences (i.e., a single nonsignificant value in a time course was treated as a new cluster), with eight clusters emerging in each object value condition. Second, following the recommendation that an effect size of *d* = 0.4 comprises a good estimate of the smallest effect size of interest in psychological research (Brysbaert, 2019), a cluster analysis was conducted on the effect sizes associated with the *p* values.

**Fig. 3:**
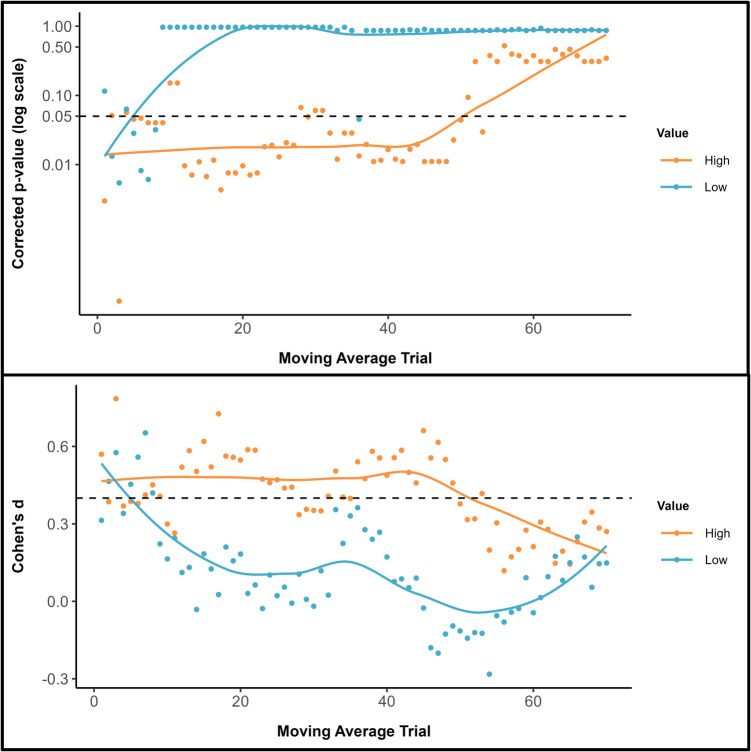
Upper panel: Adjusted *p* values for the self-prioritization effect are presented on a logarithmic scale, with a locally estimated scatterplot smoothing (LOESS) curve superimposed to illustrate trends over the trial sequence. The dashed horizontal line marks the standard significance threshold (*p* = .05), allowing for a quick visual assessment of which trials and conditions showed statistically significant latency differences after applying an FDR correction. Lower panel: Effect sizes (Cohen’s *d*) associated with the p-values. The dashed horizontal line marks the smallest effect size of interest (*d* = 0.4)

As can be seen in Fig. 3 (upper & lower panels), self-prioritization was more enduring for high-value compared with low-value items, and effect sizes were larger for the former than latter stimuli. Interestingly, whereas personal possession only benefitted the classification of low-value items during the early phases of the experiment, high-value objects were advantaged until toward the end of the testing session.

### Discussion

Confirming our hypothesis, self-prioritization was moderated by the reward value of the to-be-judged objects (Lee et al., 2023; Sui et al., 2012; Yankouskaya et al., 2023). Based on an average moving window analysis, the starting point moving average was faster for self-associated compared with friend-associated objects. Additionally, as the task progressed, response latencies decreased more rapidly for friend-owned compared with self-owned items. In terms of the time course of decisional bias, self-prioritization was more pronounced and persistent for high-value compared with low-value objects. Indeed, for low-value objects, the benefits of self-association were relatively short-lived, emerging only during the initial stages of the task. In contrast, high-value objects were prioritized until toward the end of the experiment, at which point self-bias was eliminated. Underlining the benefits of this analytical approach, these temporal effects were obscured when the data were aggregated across the entire testing session (Golubickis et al., 2018, 2019).

Given the reported results, the motivation for our next experiment was to attempt to replicate the findings observed in Experiment 1 using different stimuli. Specifically, rather than employing objects with established value (e.g., rubies & pebbles), in Experiment 2, participants possessed symbolic stimuli (i.e., cryptocurrencies) that varied with respect to their assigned monetary worth (i.e., high vs. low). Despite the shift in object type, we expected self-prioritization to be more marked and persistent for high-value compared with low-value stimuli.

## Experiment 2: Owning cryptocurrencies

### Method

#### Participants and design

Forty participants (16 women, 24 men: *M*_age_ = 25.13 years, *SD* = 2.60), with normal or corrected-to-normal visual acuity completed the experiment. Data collection was conducted online using Prolific Academic (http://www.prolific.co), with each participant receiving compensation at the rate of £10 (~$13) per hour. Informed consent was obtained from participants prior to the commencement of the experiment and the protocol was reviewed and approved by the Ethics Committee at the School of Psychology, University of Aberdeen. The experiment had a 2 (owner: self vs. friend) × 2 (symbol value: high vs. low) repeated-measures design. The power calculation was as in Experiment 1.

#### Stimulus materials and procedure

**Fig. 4:**
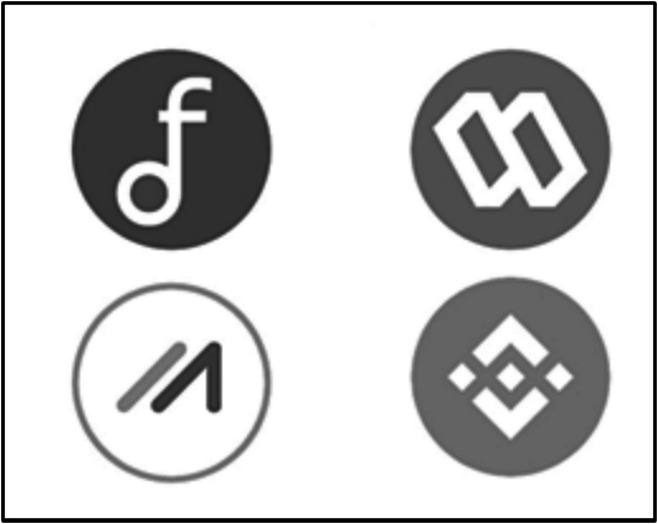
Cryptocurrencies owned by self and best friend

The procedure was as in Experiment 1, but with a single modification. On this occasion, self and friend ostensibly owned cryptocurrencies that varied in monetary value. Prior to the beginning of the task, participants were shown symbols pertaining to four cryptocurrencies (see Fig. 4). Underneath each symbol was a number denoting the value of the currency (i.e., £300 or £1). On screen, participants were then informed which two cryptocurrencies were owned-by-self and which two were owned-by-friend. Crucially, self and friend were each assigned a high- and a low-value cryptocurrency, with symbols and values counterbalanced across the sample. All participants reported that the presented cryptocurrencies were unfamiliar. On completion of the task, participants were debriefed and thanked.

### Results

#### Aggregated analysis

The exclusion criteria were as in Experiment 1. Two participants (men) were removed for failing to follow the instructions, and outlier screening eliminated less than 1% of the total number of trials (Golubickis et al., 2018). A Shapiro–Wilk test indicated that the RTs were normally distributed (*W* = .968, *p* = .334). Together with response accuracies, participants’ mean correct RTs were submitted to a 2 (owner: self vs. friend) × 2 (symbol value: high vs. low) repeated-measures ANOVA. Analysis of the RTs revealed a main effect of owner, *F*(1, 37) = 23.27, *p* < .001, η_p_^2^ = .39, and a significant Owner × Symbol Value interaction, *F*(1, 37) = 4.72, *p* = .036, η_p_^2^ = .11 (see Table 2). Follow-up analyses yielded a significant self-prioritization effect for both high-value, *t*(37) = 4.88, *p* < .001, *d*_z_ = 0.79, and low-value, *t*(37) = 2.34, *p* = .025, *d*_z_ = 0.38, symbols.

**Table 2:** Mean RT (ms) and accuracy (%) as a function of owner and symbol value (Exp. 2)

	Symbol value	
Owner	High	Low
*RT*		
Self	445 (50)	455 (48)
Friend	465 (51)	465 (49)
*Accuracy*		
Self	96 (3)	96 (3)
Friend	96 (3)	97 (2)

Standard deviation in parentheses.

Analysis of response accuracies revealed only a significant Owner × Symbol Value interaction, *F*(1, 37) = 4.22, *p* = .047, η_p_^2^ = .10 (see Table 2). Follow-up analyses indicated that, for low-value symbols, responses were more accurate to friend-owned compared with self-owned items, *t*(37) = −2.76, *p* = .009, *d*_z_ = 0.45. No such significant effect was observed for high-value symbols, *t*(37) = 0.05, *p* = .957, *d*_z_ = 0.01.

#### Moving window analysis

**Fig. 5:**
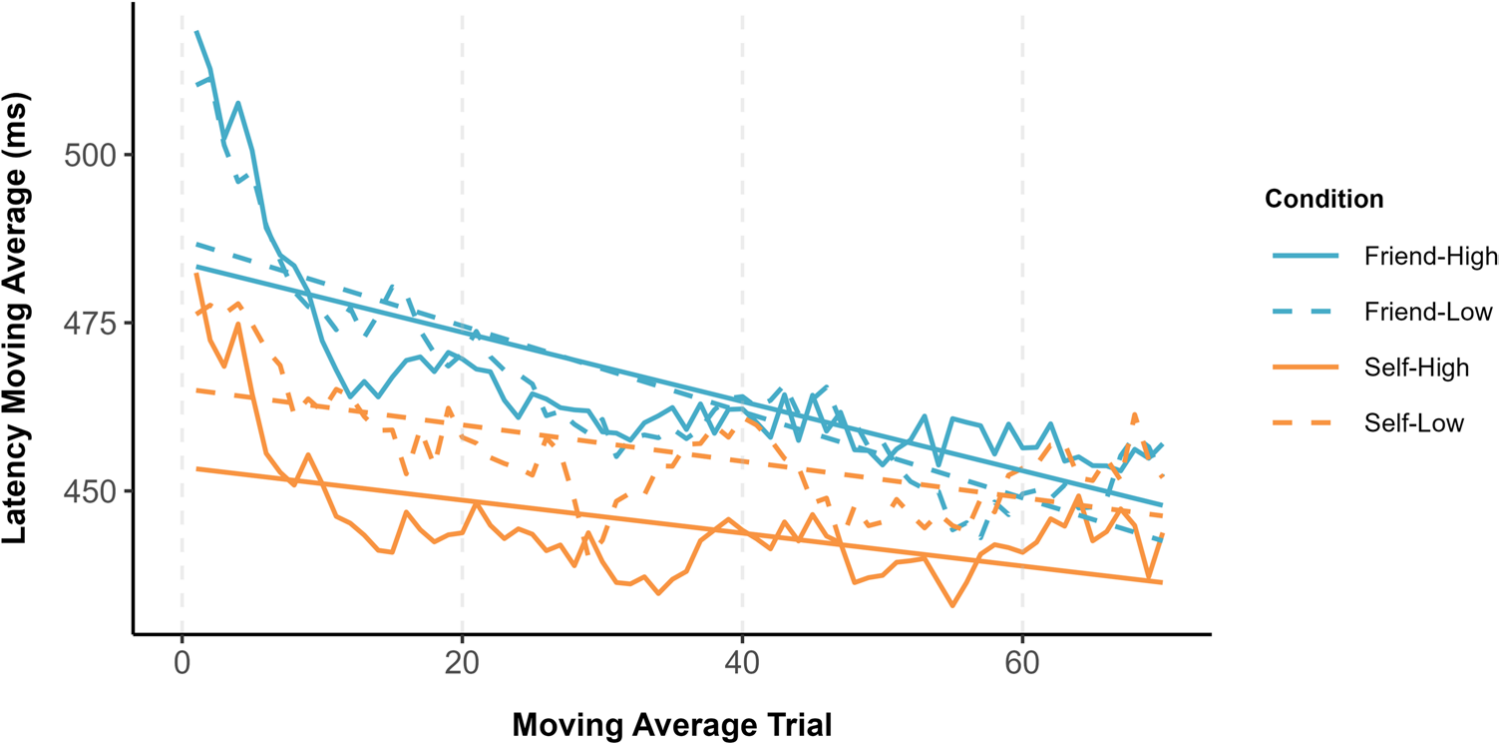
Moving window latency averages as a function of owner and symbol value

Following Experiment 1, a moving average approach (window size of 10 trials) was applied to the RTs. As previously, additional analyses were conducted using window sizes of five and 15 trials (see Supplementary Information). Linear models were fitted for each experimental condition which cumulatively explained 50.6% of the variance (see Fig. 5). The intercepts and slopes derived from the linear models were submitted to a 2 (owner: self vs. friend) × 2 (symbol value: high vs. low) repeated-measures ANOVA. Analysis of the intercepts revealed only a main effect of owner, *F*(1, 37) = 31.35, *p* < .001, η_p_^2^ = .46, such that the starting point of the moving average was faster for self-owned (*M* = 459 ms, *SE* = 10 ms) compared with friend-owned (*M* = 485 ms, *SE* = 10 ms) items. Similarly, the analysis of slope coefficients again yielded only a significant main effect of owner, *F*(1, 37) = 15.24, *p* < .001, η_p_^2^ = .29, indicating that the rate at which the moving average decreased over trials was higher for friend-owned (*M* = −.58, *SE* = .13) compared with self-owned (*M* = −.26, *SE* = .13) symbols.

**Fig. 6:**
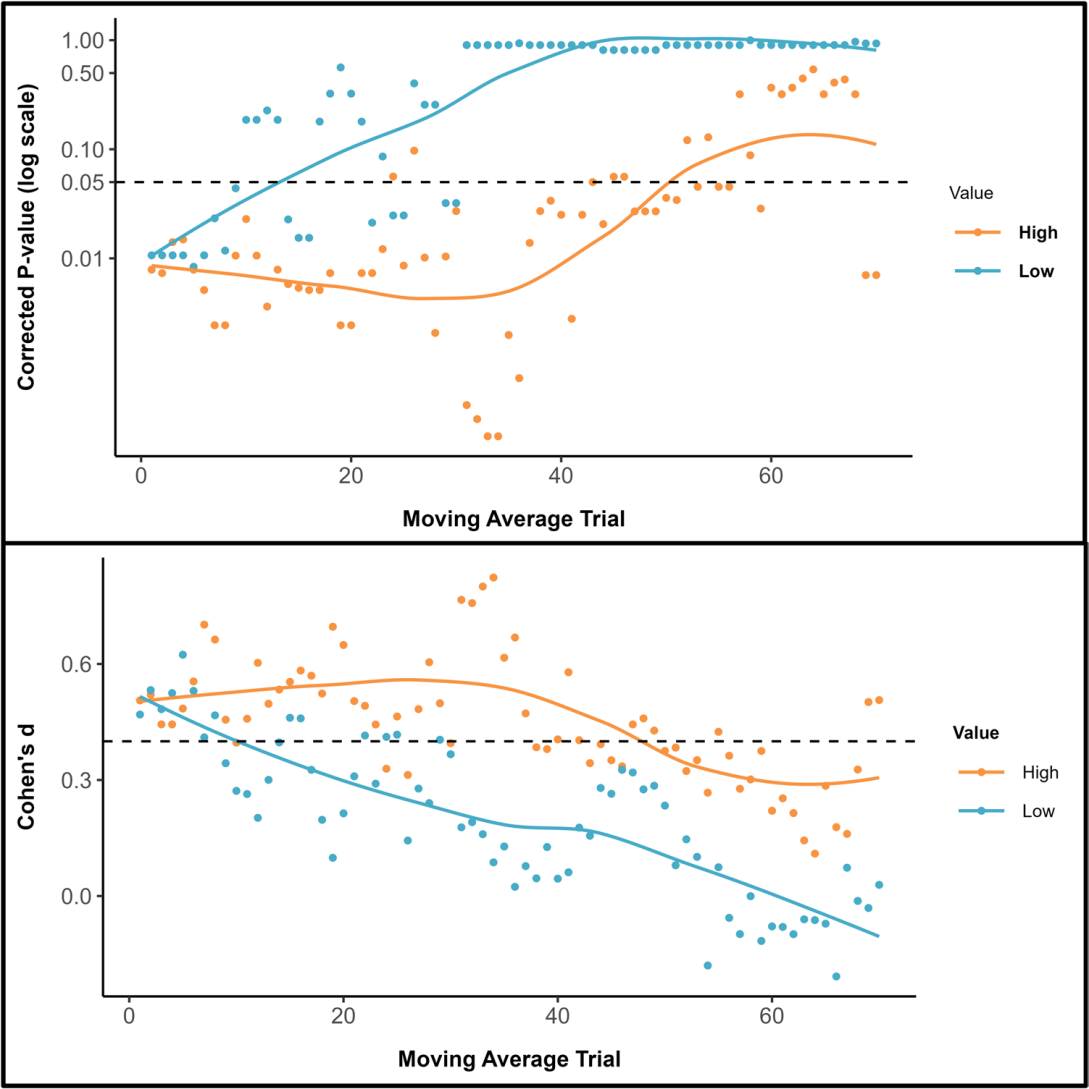
Upper Panel: Adjusted *p* values for the self-prioritization effect are presented on a logarithmic scale, with a locally estimated scatterplot smoothing (LOESS) curve superimposed to illustrate trends over the trial sequence. The dashed horizontal line marks the standard significance threshold (*p* = .05), allowing for a quick visual assessment of which trials and conditions showed statistically significant latency differences after applying an FDR correction. Lower panel: Effect sizes (Cohen’s *d*) associated with the *p* values. The dashed horizontal line marks the smallest effect size of interest (*d* = 0.4)

As previously, cluster analyses of the self-prioritization effect for both symbol value conditions was performed with *p* values (FDR corrected) and associated effect sizes plotted across the moving average windows (see Fig. 6). While for high-value symbols seven clusters were detected, 13 clusters were observed for low-value symbols. As can be seen in Fig. 6 (upper and lower panels), self-prioritization was more persistent for high-value compared with low-value stimuli and effect sizes were larger for the former than latter items. Indeed, whereas personal possession only benefitted the classification of low-value items during the very early phases of the experiment, high-value symbols were advantaged until toward the end of the testing session, at which point self-prioritization was eliminated. Additional analyses using window sizes of five and 15 yielded comparable results (see Supplementary Information).

### Discussion

Despite using different stimuli (i.e., cryptocurrencies), the current results corresponded with the results reported in Experiment 1. As previously, the starting point moving average was faster for self-associated compared with friend-associated items and, as the task progressed, response latencies decreased more rapidly for the latter than former stimuli. Crucially, in terms of the temporal characteristics of decisional bias, self-prioritization was more pronounced and enduring for high-value compared with low-value cryptocurrencies. Replicating Experiment 1, the benefits of personal possession for low-value items were transitory, emerging only during the initial stages of the task. In contrast, high-value items were prioritized until near the end of the testing session.

A potential limitation with the current findings concerns how variability in self-bias could be driven by differences in the ease with which information pertaining to self and friend was acquired (Golubickis & Macrae, 2022; Jalalian et al., 2023, 2024b).  In keeping with prior work on the topic, participants performed the object-classification task following a brief period in which they were provided with the specific target–cryptocurrency–value associations (Golubickis et al., 2018). If, however, self-related and friend-related stimulus associations were not acquired equally during this time, it is possible that differences in decisional bias during the early stages of the object-classification task reflected the contribution of an additional learning effect. Accordingly, to address this matter, in our next experiment participants first performed an explicit learning phase in which they worked out which cryptocurrencies (high-value & low-value) belonged to self and friend (Jalalian et al., 2023). Learning was established when participants achieved sufficient levels of accuracy across all the symbols (Frank et al., 2004, 2007), at which point they completed the object-classification task. As previously, it was expected that self-prioritization would be more pronounced and persistent for high-value compared with low-value stimuli.

## Experiment 3: Learning about and owning cryptocurrencies

### Method

#### Participants and design

Forty participants (28 women, 12 men: *M*_age_ = 24.75, *SD* = 2.62), with normal or corrected-to-normal visual acuity completed the experiment. The experiment was conducted online using Prolific Academic (http://www.prolific.co), with each participant receiving compensation at the rate of £10 (~$13) per hour. Informed consent was obtained from participants prior to the commencement of the experiment and the protocol was reviewed and approved by the Ethics Committee at the School of Psychology, University of Aberdeen. The experiment had a 2 (owner: self vs. friend) × 2 (symbol value: high vs. low) repeated-measures design. The power calculation was as in Experiments 1 and 2.

#### Stimulus materials and procedure

The procedure mirrored that of Experiment 2, but with a key modification concerning how participants acquired the target–cryptocurrency–value associations. Specifically, instead of explicitly instructing participants who owned what, Experiment 3 introduced a learning phase in which the target–symbol associations had to be learned to criterion prior to the object classification (Jalalian et al., 2023). Utilizing the same stimuli as Experiment 2, participants were informed they were required to learn, based on feedback provided, which cryptocurrencies were owned-by-self and which were owned-by-friend. On each trial, a symbol appeared in the center of the computer screen and participants selected, among four response options (i.e., You-£300, You-£1, Friend-£300, Friend-£1), to whom the cryptocurrency belonged. Responses were made by a mouse click on the relevant option, all of which appeared underneath the symbols. Immediate feedback was provided on screen after each choice indicating the accuracy of the response (i.e., green ‘Correct’ or red ‘Incorrect’).

The learning phase comprised six blocks of eight trials, with each cryptocurrency appearing twice per block. Participants continued the learning phase until they completed an entirely error free block of trials (i.e., eight out of eight trials). Once this criterion was met, the object-classification task commenced. If participants did not acquire the relevant associations within 6 blocks, the learning phase concluded. As in Experiment 2, the cryptocurrencies and monetary values were counterbalanced across the sample and, prior to the experiment, all participants reported that the symbols were unfamiliar. Upon completion of the task, participants were debriefed and thanked.

### Results

Participants successfully learned the stimulus associations to criterion in an average of 2.57 blocks (*SD* = 0.99): 10% of participants achieved criterion within one block; 41% within two blocks; 26% within three blocks; 15% within four blocks; and 8% within five blocks.

#### Aggregated analysis

The exclusion criteria were as in Experiment 1. Two participants (women) were removed for failing to follow the instructions, and outlier screening eliminated less than 1% of the total number of trials (Golubickis et al., 2018). A Shapiro–Wilk test indicated that the RTs were normally distributed (*W* = .968, *p* = .353). Participants’ mean correct RTs and response accuracies were submitted to a 2 (owner: self vs. friend) × 2 (symbol value: high vs. low) repeated-measures ANOVA. Analysis of the RTs revealed a main effect of owner, *F*(1, 37) = 24.83, *p* < .001, η_p_^2^ = .40, and a significant Owner × Symbol Value interaction, *F*(1, 37) = 6.49, *p* = .015, η_p_^2^ = .15 (see Table 3). Follow-up analyses yielded a significant self-prioritization effect for both high-value, *t*(37) = 5.85, *p* < .001, *d*_z_ = 0.95, and low-value, *t*(37) = 2.71, *p* = .010, *d*_*z*_ = 0.44, symbols.

**Table 3:** Mean RT (ms) and accuracy (%) as a function of owner and symbol value (Exp. 3)

	Symbol value	
Owner	High	Low
*RT*		
Self	451 (68)	462 (70)
Friend	477 (75)	475 (70)
*Accuracy*		
Self	96 (3)	95 (3)
Friend	95 (5)	95 (5)

Standard deviation in parentheses.

Analysis of response accuracies revealed only a significant Owner × Symbol Value interaction, *F*(1, 37) = 4.19, *p* = .048, *η*_*p*_^*2*^ = .10 (see Table 3). Follow-up analyses revealed no significant effects.

#### Moving window analysis

**Fig. 7:**
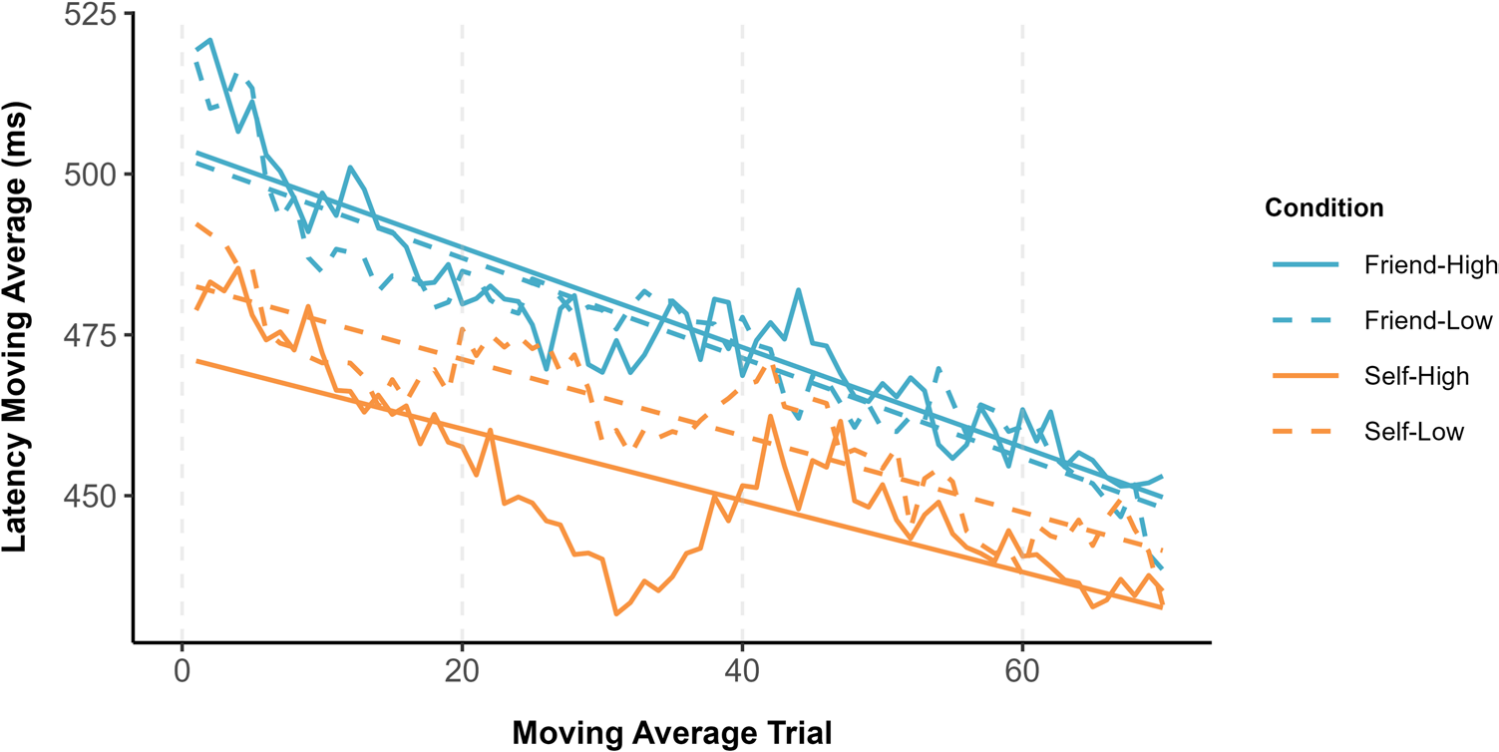
Moving window latency averages as a function of owner and symbol value

Following the previous experiments, a moving average approach (window size of 10 trials) was applied to the RTs. Linear models were fitted for each experimental condition which cumulatively explained 79.7% of the variance (see Fig. 7). The intercepts and slopes derived from the linear models were submitted to a 2 (owner: self vs. friend) × 2 (symbol value: high vs. low) repeated-measures ANOVA. Analysis of the intercepts revealed only a main effect of owner, *F*(1, 37) = 17.17, *p* < .001, η_p_^2^ = .32, such that the starting point of the moving average was faster for self-owned (*M* = 476 ms, *SE* = 13 ms) compared with friend-owned (*M* = 503 ms, *SE* = 15 ms) items. The analysis of slope coefficients yielded no significant effects.

**Fig. 8:**
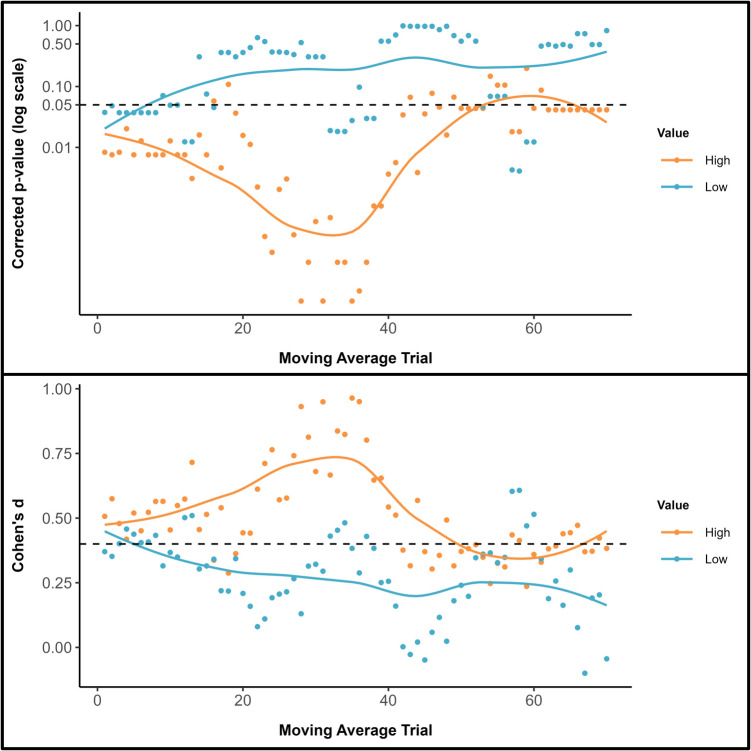
Upper panel: Adjusted *p* values for the self-prioritization effect are presented on a logarithmic scale, with a locally estimated scatterplot smoothing (LOESS) curve superimposed to illustrate trends over the trial sequence. The dashed horizontal line marks the standard significance threshold (*p* = .05), allowing for a quick visual assessment of which trials and conditions showed statistically significant latency differences after applying an FDR correction. Lower panel: Effect sizes (Cohen’s *d*) associated with the *p* values. The dashed horizontal line marks the smallest effect size of interest (*d* = 0.4)

As previously, cluster analyses of the self-prioritization effect for both symbol value conditions were performed with *p* values (FDR corrected) and associated effect sizes plotted across the moving average windows (see Fig. 8). While for high-value symbols 17 clusters were detected, 14 clusters were observed for low-value symbols. As can be seen in Fig. 8 (upper and lower panels), self-prioritization was more persistent for high-value compared with low-value stimuli and effect sizes were larger for the former than latter items. Indeed, whereas personal possession benefitted the classification of low-value items only during the very early phases of the experiment, high-value symbols were advantaged until toward the end of the testing session, at which point self-prioritization was eliminated. Additional analyses using window sizes of five and 15 yielded comparable results (see Supplementary Information).

### Discussion

Replicating Experiments 1 and 2, comparable effects emerged when the learning of self-related and friend-related cryptocurrencies were equated prior to the performance of the object-classification task. First, the starting point moving average was faster for self-associated compared with friend-associated symbols. Second, self-prioritization was more pronounced and persistent for high-value compared with low-value stimuli. While the benefit of personal possession for low-value items was fleeting, emerging only during the early stages of the object-classification task, for high-value items this advantage endured until near the end of the testing session.

## General discussion

Across three experiments employing objects with both established and assigned worth, a consistent pattern of effects was observed. Reward value moderated the magnitude and duration of self-bias (Sui et al., 2016, 2023; Yankouskaya et al., 2020, 2023). Rather than reflecting a fixed product of decisional processing (Humphreys & Sui, 2016; Sui & Humphreys, 2015, 2017), stimulus prioritization was sensitive to the value (i.e., high vs. low) of objects and when in the testing session judgments-of-ownership were probed. Notably, whereas low-value stimuli benefitted from personal association during the early phases of the task, high-value items were prioritized for longer, with self-bias disappearing only toward the end of the experiment. During an object-classification task, these findings illustrate the advantages of an analytical approach that examines the temporal profile of self-bias (cf. Falbén et al., 2020; Golubickis et al., 2018, 2019, 2021a).

### The stability of self-bias

A core assumption of influential accounts of social cognition is that self-prioritization is a stable product of decision-making (Baumeister, 1998). Regardless of people’s processing concerns, properties of the to-be-judged stimuli, or the characteristics of the task environment, self-bias is an obligatory facet of mental life (Humphreys & Sui, 2016; Sui & Humphreys, 2015, 2017). Contesting this account, however, recent work has revealed self-prioritization to be inherently pliable, arising only under certain conditions (Constable et al., 2019; Dalmaso et al., 2019; Falbén et al., 2019; Jalalian et al., 2023; Siebold et al., 2015; Stein et al., 2016; Svensson et al., 2022; Woźniak & Knoblich, (Woźniak et al., 2022)). Corroborating this viewpoint, here we identified another important determinant of decisional bias, time.

In each of the reported experiments, not only did stimulus prioritization fluctuate over the duration of the object-classification task, response latencies to the targets of interest varied considerably (see Figs. 2, 5, & 7). First, compared with friend-related responses, self-related responses were faster from the outset of the experiments. Second, in Experiments 1 and 2, response latencies decreased more rapidly for friend-related than self-related responses, an effect that was not observed in Experiment 3. This inconsistency can likely be traced to the introduction of the explicit learning phase in Experiment 3 in which target-object associations had to be learned to criterion prior to the object-classification task (see also Jalalian et al., 2023). Given natural differences in the ease in which self-object and friend-object linkages can be formed, the latter stimuli would have benefitted most from the learning task. Interestingly, this benefit was restricted to the rate at which response latencies decreased and had no impact on the emergence or time course of self-prioritization. Self-bias was most pronounced at the beginning of the object-classification task in all three experiments, thereby revealing a mind that is finely tuned for the processing of personally relevant material (Conway & Pleydell-Pearce, 2000; Heatherton, 2011; Heatherton et al., 2004; Humphreys & Sui, 2016; Sui & Humphreys, 2015, 2017).

Shaping the time course of self-bias was the reward-value of the items. Moreover, whether the stimuli had established (i.e., Exp. 1) or assigned (i.e., Exps. 2, 3) worth, a consistent pattern of effects was observed. Whereas low-value items were prioritized during the initial stages of the object-classification task, for high-value items self-bias persisted until near the end of the testing session. These differences in the profile of self-bias may reflect the rate at which participants habituated to stimuli with varying levels of reward value (Rankin et al., 2009). Although advantageous (i.e., self-enhancing) to link both high- and low-value items with the self (Alicke & Sedikides, 2009), these benefits nonetheless diminish as a function of stimulus repetition. Crucially, however, by engaging attention for longer (Berkman et al., 2017; Humphreys & Sui, 2016; Northoff & Hayes, 2011; Sui & Humphreys, 2015, 2017), highly rewarding stimuli have a greater resistance to habituation, thus generate more persistent self-prioritization effects. Of course, to maintain processing flexibility in an ever-changing stimulus world, even high-value items eventually lose their potency (i.e., positivity) following repeated presentation and classification.

## Self-prioritization revisited

Using aggregated data, previous research has shown self-prioritization to be eliminated under various conditions (Golubickis & Macrae, 2023). For example, self-bias is conditional upon goal-relevance, such that self-owned (vs. friend-owned) objects are only prioritized when the task instructions explicitly draw attention to target-item associations in memory (e.g., Is the object owned-by-self or owned-by-friend?). When, in contrast, items are judged on a dimension unrelated to ownership (e.g., Did the stimulus appear to the left or right of fixation?), self-prioritization is abolished (Constable et al., 2019). Similarly, when no information is provided about how many self-related and friend-related items will be encountered during the task, self-prioritization emerges. When, however, participants are told equal numbers of self-owned and friend-owned items will be presented, decisional bias is eliminated (Falbén et al., 2020). Of course, the eradication of self-bias in these experiments was based on responses (i.e., RT_self_ vs. RT_friend_) that were averaged across the entire testing session. Given the current findings, it remains possible that self-owned objects may have been prioritized during the initial stages of the decision-making task, even when items were goal-irrelevant and the frequency of stimulus presentation was known. A temporal analysis will be required to establish if this was in fact the case.

Beyond the effects exerted by variations in the experimental instructions, perceiver-related factors may also influence the temporal profile of self-prioritization, but interestingly toward the end of the testing session. Consider, for example, the effects of brief mindfulness-based meditation on the emergence of self-bias. Impacting the character of self-referential processing (Bishop et al., ( 2004); Brown & Ryan, 2003; Hölzel et al., 2011; Kabat-Zinn, 2003), short-term (e.g., 5 min) episodes of mindfulness meditation have been shown to eliminate self-bias, both in classic social-psychological experimental contexts (e.g., spotlight effect; Golubickis et al., 2016) and object-classification tasks (Golubickis et al., 2023). Applying a computational analysis to establish the pathway through which debiasing occurs, Golubickis et al. (2023) demonstrated that mindfulness-based meditation abolished the egocentric response-related strategy that underpins self-bias during binary decision-making. Specifically, following a brief period of meditation, less evidence was required to select self-relevant compared with friend-relevant responses (Falbén et al., 2020; Golubickis et al., 2018, 2019). Crucially, however, for how long decisional processing was inoculated from egocentrism remains unknown. A productive application of the current analytical approach will therefore be to establish if, following a brief meditative episode, self-bias reemerges toward the latter stages of the testing session. Combined with different dosages of mindfulness meditation, work of this kind will inform understanding of the longevity of decisional debiasing (Papies et al., 2012, 2015).

Here, we demonstrated that even low-value objects briefly benefitted from prior self-association. Of course, while low in worth, such items can nevertheless still be construed in a positive manner. But what about material for which this is not the case—for example, items that directly challenge the self-concept (Baumeister, 1998)? An emerging literature has identified valence to be an important determinant of self-prioritization, such that positive stimuli (e.g., desirable posters) reliably trigger self-bias, but negative stimuli (e.g., undesirable posters) do not (Golubickis et al., 2021a, b; Hu et al., 2020; Lee et al., 2023; Vicovaro et al., 2022). A favoured explanation for this asymmetry resides in the associations formed in memory between self and items that vary in attractiveness—hence, connotations for the self-concept (Ye & Gawronski, 2016). Whereas self-object linkages are bolstered for desirable items, inhibitory connections are formed between the self and undesirable stimuli, a tactic that ultimately protects the self-concept from threat (Alicke & Sedikides, 2009). This then raises an interesting question. Considering the time course of self-bias, might even undesirable self-relevant stimuli be prioritized, at least temporarily, during the initial stages of an object-classification task (i.e., self-relevance initially trumps stimulus negativity)? To further elucidate the temporal characteristics of self-prioritization, this possibility merits future investigation.

Of theoretical interest is how exactly reward-value impacts the emergence of self-bias (Yankouskaya et al., 2020, 2023). Most would agree that self-enhancement motivation (i.e., the drive to view self in a favourable light) plays a pivotal role in this regard. By associating self with positive, desirable, and high-value stimuli (vs. negative, undesirable, and low-value stimuli), a flattering self-image can be maintained (Sedikides & Strube, 1997). A basic cognitive mechanism through which this self-enhancement can be realized resides in the efficiency of stimulus processing during decision-making (Golubickis et al., 2017; Sui et al., 2023). Applying computational modeling to elucidate the operations driving self-prioritization, previous research has demonstrated that decisional evidence is extracted more rapidly when stimuli have positive compared with negative implications for the self-concept (Golubickis et al., 2021a, b; Hu et al., 2020). Mirroring this effect, a comparable bias likely underpinned the effects of reward-value observed in the current investigation, albeit with the added influence of time as a determinant of processing efficiency.

Aligning with work demonstrating that undesirable posters are classified more quickly when associated with friend than self (i.e., friend-prioritization; Golubickis et al., 2021a, b), here we found that low-value stimuli were judged more accurately when linked with the former compared with the latter target (i.e., Exps. 1 & 2). Furthermore, inspection of Figs. 2, 5, and 7 reveals interesting differences in the pattern of response latencies for friend-related stimuli as a function of stimulus type. Whereas for friend-owned objects with established value (i.e., rocks) response latencies were faster for low-value than high-value items, this pattern was not observed for stimuli with assigned worth (i.e., cryptocurrencies). This highlights the important influence that stimulus properties exert on decisional processing (Falbén et al., 2020; Golubickis et al., 2021a, b; Hu et al., 2020; Sui et al., 2012), with attendant implications for theoretical accounts of self-function (Golubickis & Macrae, 2023; Humphreys & Sui, 2016; Sedikides & Strube, 1997). Notably, by associating certain classes of stimuli (e.g., undesirable posters, worthless pebbles) more strongly with targets other than the self, this suggests a complementary pathway through which a favourable self-image can be established and sustained.

## Time and self-bias

In the only other research to explore the time course of self-prioritization, Jalalian et al. (2024a, b) reported significant levels of self-bias across the entirety of a shape-label matching task. Contrasting the current findings, self-bias was a persistent product of decisional processing. Basic differences between the paradigms under investigation may account for these discrepancies, with implications for theoretical accounts of self-function (Golubickis et al., 2018; Sui et al., 2012). First, unlike object-classification-tasks, responses during shape-label matching tasks do not reference specific targets (i.e., self or friend) but rather previously learned stimulus combinations (i.e., matching vs. nonmatching). Second, whereas the stimuli in shape-label matching tasks have no implications for the self-concept, in object-classification tasks they frequently do (Golubickis et al., 2021a; Hu et al., 2020; Lee et al., 2023; Vicovaro et al., 2022). These response- and stimulus-related differences, we suspect, may exert considerable influence on the time course of self-bias. When, for example, abstract stimuli (e.g., geometric shapes) are paired with various social targets (e.g., self-friend, stranger), the personal relevance of the items is the most salient aspect of the task context. This is not the case, however, in object-classification tasks where stimuli have both personal relevance and ramifications for the self-concept. Under these conditions, the duration of self-bias likely reflects the interplay between the cognitive and motivational operations that ultimately underpin self-referential processing. In other words, the temporal profile of self-prioritization reflects which material is associated with self and how and when these self-object linkages are probed (Golubickis et al., 2018; Jalalian et al., 2024a, b; Sui et al., 2012).

From a theoretical standpoint, the current findings highlight the need for models of self-function to accommodate variability in the time course of decisional bias. Dominating the intellectual landscape, the influential SAN model advances a largely static account of self-bias (Humphreys & Sui, 2016; Sui & Humphrey, (Sui et al., 2015)). Through a combination of top-down (i.e., self-relevance) and bottom-up (i.e., attentional capture) cognitive operations, personally relevant stimuli are prioritized during decisional processing. Absent from the model, however, is a nuanced description of how exactly the processes that support self-prioritization develop through time (Freeman & Ambady, 2011; Herbst & Landau, 2016; Stolier & Freeman, 2016). This oversight is limiting. As Stolier and Freeman (2016) have reported, “Dynamic and iterative models of social perception highlight the importance of evaluating the state of the cognitive system across time” (p. 355). Given the pivotal role of attention (e.g., selecting, orienting, engagement, disengagement) in the generation of self-bias, this is a pertinent observation. While some attentional processes may be deployed consistently across an entire testing session, others may not (Eysenck et al., 2007). Additionally, properties of to-be-judged stimuli or idiosyncrasies of the task set up may differentially influence specific components of attention over time (Svensson et al., 2023), with direct implications for the persistence of self-bias (Golubickis & Macrae, 2023). Given the cognitive and motivational determinants of self-function, researchers should address how decisional bias can be incorporated within dynamic process-based accounts of self-function.

Inevitably, the current investigation has several constraints on generality. Most notably, participants were all recruited from the UK. Grounded in different patterns of self-construal, cultural factors have been shown to influence self-referential processing, including the emergence of ownership effects (Han & Northoff, 2008; Kitayama & Uskul, 2011; Markus & Kitayama, 1991; Sparks et al., 2016). Whereas participants from individualistic Western cultures reliably display self-bias, their interdependent Asian counterparts are much less likely to do so. Indeed, among South-East Asians, Sparks et al. (2016) demonstrated a reversed self-prioritization effect when self was compared with a close other. Additional research is therefore required to establish how exactly cultural forces influence the effects of reward value on the time course of decisional bias. Also, in exploring the topic of interest, only a limited range of objects were employed in a task context that used response latencies to index self-bias. It therefore remains to be seen whether the current effects would generalize across other stimuli and measures that have been used to explore self-prioritization, including eye/hand movements and evoked response potentials (Constable et al., 2011; Dalmaso et al., 2019; Sui et al., 2023).

## Conclusion

Using stimuli that varied in reward value in an object-classification task, here we explored the temporal characteristics of self-prioritization (Haciahmet et al., 2023; Lu et al., 2024; Sui et al., 2023). A consistent pattern of effects was observed. Self-bias was more pronounced and persistent for high-value compared with low-value stimuli, regardless of whether the items had established (i.e., Exp. 1) or assigned (i.e., Exps. 2 & 3) worth. These findings extend understanding of self-bias in several important ways (Golubickis & Macrae, 2023; Humphreys & Sui, 2016; Sui & Humphreys, 2015, 2017). First, they underscore the value of analytical approaches that yield a fine-grained account of decisional bias. Second, they corroborate the malleability of self-prioritization. Third, they draw attention to a hitherto largely unexplored aspect of self-bias, its temporal profile. In charting the effects of personal possession on decisional processing, time matters.

## Supplementary Information

Below is the link to the electronic supplementary material.

Supplementary file1 (DOCX 559 KB)

## Data Availability

Data and materials for all experiments are publicly available at the Open Science Framework and can be accessed online (https://osf.io/w853p/).

## Footnotes

1 This analytical approach has also been favoured in research using shape-label matching tasks to explore self-prioritization (e.g., Golubickis et al., 2017, 2020; Reuther & Chakravarthi, 2017; Sui et al., 2012, 2013, 2015; Svensson et al., 2022).

## Code availability

Code is publicly available at the Open Science Framework and can be accessed online (https://osf.io/w853p/).

## Declarations

### Open practices statement

Data and materials for all experiments are publicly available at the Open Science Framework and can be accessed online (https://osf.io/w853p/). None of the experiments was preregistered.
