# Supplementary material for: Reward value shapes the time course of self-bias
Source: Mem Cognit. 2025 Aug 4;54(2):483–500. doi: 10.3758/s13421-025-01763-4 (PMC12957044; doi:10.3758/s13421-025-01763-4)

**Supplementary Material**

**Temporal Analysis Using a Moving Average Window of 5 and 15 Trials**

Given that a common window size for a moving average analysis can vary between 5% to 20% of the time series (Chatfield, 2003), to ensure that the results of the temporal analysis remained similar for moving window sizes other than 10, the analysis was repeated using window sizes of 5 and 15 trials in Experiment 1 (Figures S1 to S3), Experiment 2 (Figures S4 to S6), and Experiment 3 (Figures S7 to S9). We conducted 2 (Owner: self vs. friend) X 2 (Object/Symbol Value: high vs. low) repeated measures ANOVAs on the intercepts and slopes derived from the linear models. The results were similar for all moving latency average window sizes.

**Experiment 1: Owning Stones**

**Figure S1**

*Moving window latency averages (Expt. 1).*


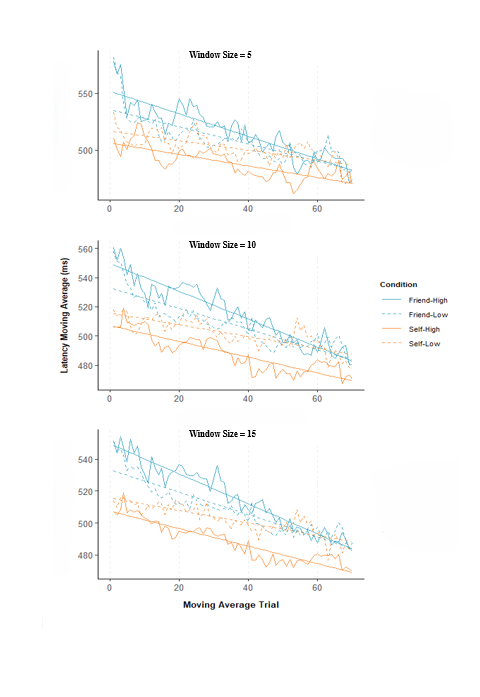


***Moving Average Analysis with Window Sizes of 5 and 15 Trials***

The intercepts and slopes derived from the linear models were submitted to a 2 (Owner: self vs. friend) X 2 (Object Value: high vs. low) repeated measures ANOVA (see Figure S1). With a window size of 5 trials, analysis of the intercepts showed only a main effect of Owner (*F*(1, 35) = 23.66, *p* < .001, *η_p_^2^* = .40), with a faster starting point average for self-owned (*M* = 512 ms, *SE* = 13 ms) compared to friend-owned (*M* = 547 ms, *SE* = 13 ms) items. Analysis of the slopes also only yielded a main effect of Owner (*F*(1, 35) = 13.77, *p* < .001, *η_p_^2^* = .28), such that the moving average decreased at a higher rate for friend-owned (*M* = -0.93 ms, *SE* = 16 ms) compared to self-owned (*M* = -0.46 ms, *SE* = 0.021 ms) stimuli.

With a window size of 15 trials, Analysis of the intercepts showed only a main effect of Owner (*F*(1, 35) = 23.30, *p* < .001, *η_p_^2^* = .39), such that the starting point average was faster for self-owned (*M* = 512 ms, *SE* = 12 ms) compared to friend-owned (*M* = 545 ms, *SE* = 13 ms) items. Analysis of the slopes also only yielded a main effect of Owner (*F*(1, 35) = 11.64, *p* = .002, *η_p_^2^* = .25), with the moving average decreasing at a higher rate for friend-owned (*M* = -0.87 ms, *SE* = 15 ms) compared to self-owned (*M* = -0.47 ms, *SE* = 0.20 ms) stimuli.

***Cluster Analysis with Window Sizes of 5 and 15 Trials***

Cluster analyses were conducted focusing on comparing moving averages of self-owned and friend-owned items for each Object Value condition. Specifically, a paired sample *t*-test was performed for each trial number, to examine the significance of the self-prioritization effect. Compared to low-value stimuli, self-prioritization persisted longer for high-value items (see Figure S2) and the effect was larger for high-value items (see Figure S3).

**Figure S2**

*Corrected p-values of the self-prioritization effect on a logarithmic scale with a locally estimated scatterplot smoothing curve overlayed to provide a succinct representation of trends across the trial sequence. The dashed horizontal line shows the conventional threshold for statistical significance (i.e., p = .05), enabling an immediate visual appraisal of the trials and conditions under which latency differences were statistically robust post-FDR correction (Expt. 1).*


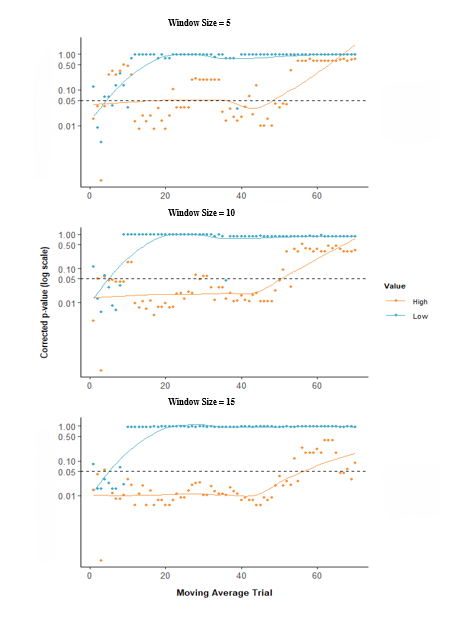


**Figure S3**

*Effect sizes (Cohen’s d) associated with the p-values. The dashed horizontal line marks the smallest effect size of interest (d = 0.4)* *(Expt. 1).*


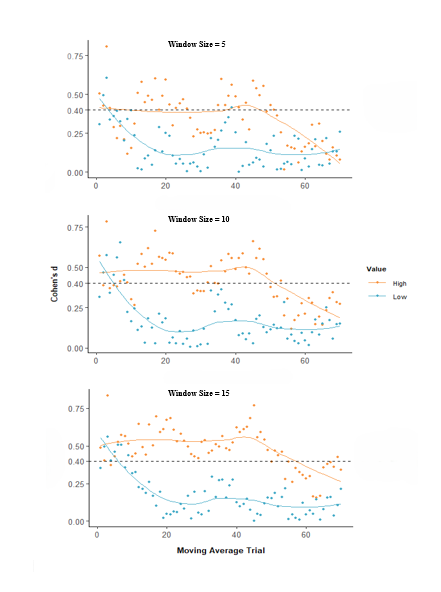


**Experiment 2: Owning Cryptocurrencies**

**Figure S4**

*Moving window latency averages (Expt. 2)*


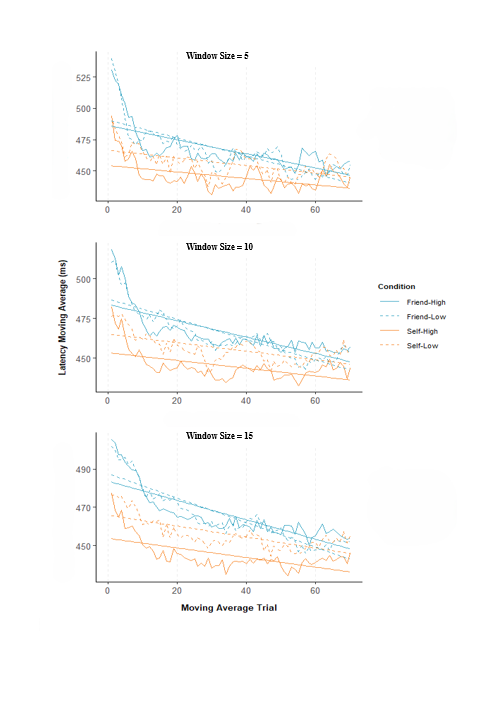


***Moving Average Analysis with Window Sizes of 5 and 15 Trials***

The intercepts and slopes derived from the linear models were submitted to a 2 (Owner: self vs. friend) X 2 (Symbol Value: high vs. low) repeated measures ANOVA (see Figure S4). As with Experiment 1, using a window size of 5 trials, analysis of the intercepts revealed only a main effect of Owner (*F*(1, 37) = 35.37, *p* < .001, *η_p_^2^* = .49), such that the starting point of the moving average was faster for self-owned (*M* = 460 ms, *SE* = 10 ms) compared to friend-owned (*M* = 488 ms, *SE* = 11 ms) items. The analysis of slope coefficients also only yielded a significant main effect of Owner (*F*(1, 37) = 18.20, *p* < .001, *η_p_^2^* = .33), indicating that the rate at which the moving average decreased over trials was higher for friend-owned (*M* = -0.64 ms, *SE* = 16 ms) compared to self-owned (*M* = -0.28 ms, *SE* = 0.14 ms) symbols.

With a window size of 15 trials, analysis of the intercepts showed only a main effect of Owner (*F*(1, 37) = 28.77, *p* < .001, *η_p_^2^* = .44), such that the starting point of the moving average was faster for self-owned (*M* = 460 ms, *SE* = 10 ms) compared to friend-owned (*M* = 485 ms, *SE* = 11 ms) items. The analysis of slope coefficients also only showed a significant main effect of Owner (*F*(1, 37) = 13.47, *p* < .001, *η_p_^2^* = .27), indicating that the rate at which the moving average decreased over trials was higher for friend-owned (*M* = -0.58 ms, *SE* = 15 ms) compared to self-owned (*M* = -0.27 ms, *SE* = 14 ms) symbols.

***Cluster Analysis with Window Sizes of 5 and 15 Trials***

Cluster analyses were performed to examine the significance of the self-prioritization effect throughout the task. As with the findings when using a window size of 10 trials, self-prioritization persisted longer for high-value (vs. low-value) items (see Figure S5), with a larger effect size for high-value items (see Figure S6).

**Figure S5**

*Corrected p-values of the self-prioritization effect on a logarithmic scale with a locally estimated scatterplot smoothing curve overlayed to provide a succinct representation of trends across the trial sequence. The dashed horizontal line shows the conventional threshold for statistical significance (i.e., p = .05), enabling an immediate visual appraisal of the trials and conditions under which latency differences were statistically robust post-FDR correction (Expt. 2).*


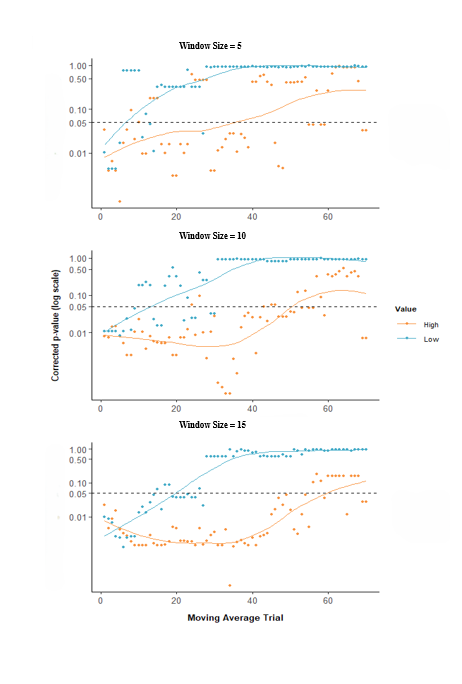


**Figure S6**

*Effect sizes (Cohen’s d) associated with the p-values. The dashed horizontal line marks the smallest effect size of interest (d = 0.4) (Expt. 2).*

*
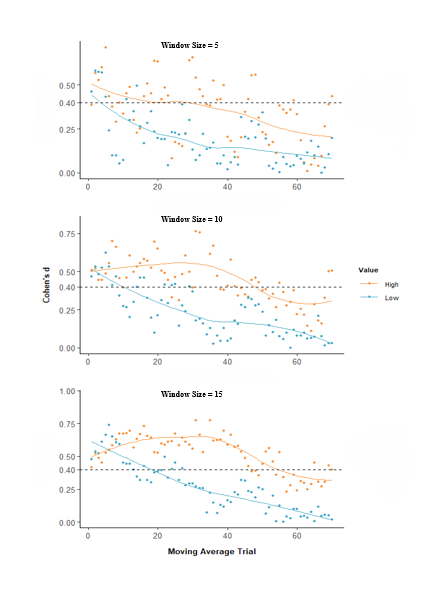
*

**Experiment 3: Learning About and Owning Cryptocurrencies**

**Figure S7**

*Moving window latency averages (Expt. 3)*

*
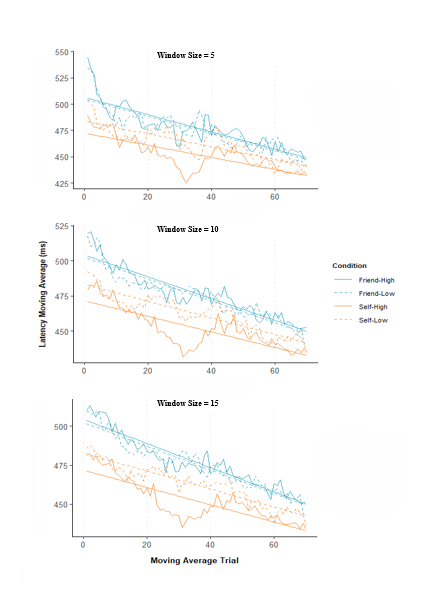
*

***Moving Average Analysis with Window Sizes of 5 and 15 Trials***

The intercepts and slopes derived from the linear models were submitted to a 2 (Owner: self vs. friend) X 2 (Object Value: high vs. low) repeated measures ANOVA (see Figure S7). Similar to Experiments 1 and 2, with a window size of 5 trials, analysis of the intercepts showed only a main effect of Owner (*F*(1, 37) = 18.05 , *p* < .001, *η_p_^2^* = .33), with a faster starting point average for self-owned (*M* = 476.68 ms, *SE* = 10 ms) compared to friend-owned (*M* = 505.27 ms, *SE* = 11 ms) items. Analysis of the slopes yielded no significant effects.

Using a 15-trial window size, analysis of the intercepts only showed a main effect of Owner *F*(1, 37) = 18.13, *p* < .001, *η_p_^2^* = .33), such that the starting point of the moving average was faster for self-owned (*M* = 503.03 ms, *SE* = 10 ms) compared to friend-owned (*M* = 485 ms, *SE* = 11 ms) items. Analysis of the slopes yielded no significant effects.

***Cluster Analysis with Window Sizes of 5 and 15 Trials***

Cluster analyses were performed to examine the significance of the self-prioritization effect throughout the task. As with the findings when using a window size of 10 trials, self-prioritization persisted longer for high-value (vs. low-value) items (see Figure S8), with a larger effect size for high-value items (see Figure S9).

**Figure S8**

*Corrected p-values of the self-prioritization effect on a logarithmic scale with a locally estimated scatterplot smoothing curve overlayed to provide a succinct representation of trends across the trial sequence. The dashed horizontal line shows the conventional threshold for statistical significance (i.e., p = .05), enabling an immediate visual appraisal of the trials and conditions under which latency differences were statistically robust post-FDR correction (Expt. 3).*


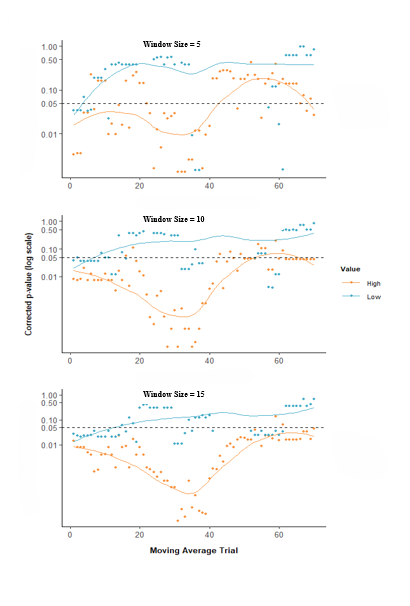


**Figure S9**

*Effect sizes (Cohen’s d) associated with the p-values. The dashed horizontal line marks the smallest effect size of interest (d = 0.4) (Expt. 3).*


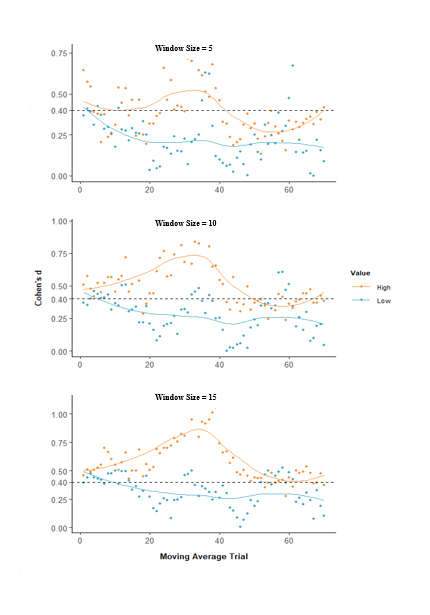

Supplement: Supplementary file 1 — Supplementary file1 (DOCX 559 KB) [file 13421_2025_1763_MOESM1_ESM.docx]
